# Rural-metropolitan health differential for young persons with eating disorders referred for specialist treatment

**DOI:** 10.1186/2050-2974-2-S1-O55

**Published:** 2014-11-24

**Authors:** Jeremy Alman, Kimberley Hoiles, Hunna Watson, Sarah Egan, Matthew Hamilton, Julie McCormack, Julie Potts, David A Forbes, Chloe Shu

**Affiliations:** 1 School of Psychology and Speech Pathology, Division of Health Sciences, Curtin University, Perth, Australia; 2 Eating Disorders Program, Specialized Child and Adolescent Mental Health Service, Child and Adolescent Health Service, Perth, Australia; 3 UNC Center of Excellence for Eating Disorders, University of North Carolina, Chapel Hill, United States; 4 School of Paediatrics and Child Health, Faculty of Medicine, Dentistry and Health Sciences, The University of Western Australia, Perth, Australia

## Objective

The aim was to explore associations between residing in a rural area and clinical characteristics of children and adolescents with eating disorders presenting to a specialist eating disorders program.

## Method

The data source was the Helping to Outline Paediatric Eating Disorders (HOPE) Project registry (N ~ 1000), a prospective ongoing registry study comprising consecutive paediatric tertiary eating disorder referrals. The sample (N = 399) comprised children and adolescents presenting with a DSM-5 eating disorder, with ages ranging from 8 to 16 years (M = 14.49, 92% female).

## Results

Consistent with the hypotheses, living in a rural area was associated with a lower body mass index z-score, and a higher likelihood of medical complications at intake assessment. Contrary to our hypothesis, eating pathology and living in a rural area were negatively associated. No relationship was observed between living in a rural area and duration of illness or greater percentage of bodyweight lost.

## Conclusions

The results suggest that living in a rural area and being a greater distance from specialist services is associated with more severe malnutrition and medical complications by the time the young person and their family obtain specialist care. These findings have implications for service planning and provision for rural communities. The modifications to service delivery in the study setting will be described.

This abstract was presented in the **Service Initiatives: Child and Adolescent** stream of the 2014 ANZAED Conference.

